# Distinction between 2′- and 3′-Phosphate Isomers of a Fluorescent NADPH Analogue Led to Strong Inhibition of Cancer Cells Migration

**DOI:** 10.3390/antiox10050723

**Published:** 2021-05-04

**Authors:** Raoul Manuel, Michelle de Souza Lima, Sébastien Dilly, Sylvain Daunay, Patricia Abbe, Elodie Pramil, Stéphanie Solier, Fabienne Guillaumond, Sarah-Simha Tubiana, Alexandre Escargueil, João Antonio Pêgas Henriques, Nathalie Ferrand, Irène Erdelmeier, Jean-Luc Boucher, Gildas Bertho, Israel Agranat, Stéphane Rocchi, Michèle Sabbah, Anny Slama Schwok

**Affiliations:** 1 Cancer Biology and Therapeutics Team, INSERM, UMR_S 938, Centre de Recherche Saint-Antoine, Sorbonne Université, F-75012 Paris, France; raoul.manuel@inserm.fr (R.M.); michelle.cacoal@gmail.com (M.d.S.L.); Sebastien.Dilly@gmail.com (S.D.); elodie.pramil@hotmail.com (E.P.); alexandre.escargueil@inserm.fr (A.E.); nathalie.ferrand@inserm.fr (N.F.); michele.sabbah@inserm.fr (M.S.); 2 Innoverda, Biopark Villejuif, F-94800 Villejuif, France; sylvain.daunay@innoverda.com (S.D.); irene.erdelmeier@innoverda.com (I.E.); 3 Centre Méditerranéen de Médecine Moléculaire (C3M), INSERM U1065, Team 12, F-06204 Nice, France; Patricia.Abbe@unice.fr (P.A.); Stephane.ROCCHI@univ-cotedazur.fr (S.R.); 4 Gustave Roussy Cancer Center, INSERM U1170, F-94805 Villejuif, France; soliersteph@yahoo.fr; 5 Centre de Recherche en Cancérologie de Marseille (CRCM), INSERM U1068, Aix-Marseille Univ., CNRS, UMR 7258, Institut Paoli-Calmettes, F-13288 Marseille, France; fabienne.guillaumond@inserm.fr (F.G.); sarah-simha.tubiana@inserm.fr (S.-S.T.); 6 Departamento de Biofísica/Centro de Biotecnologia, Universidade Federal Do Rio Grande Do Sul (UFRGS), Porto Alegre 90040-060, Brazil; pegas.henriques@gmail.com; 7 Graduate Program in Biotechnology, Universidade do Vale do Taquari—Univates, Lajeado 95900-000, Brazil; 8 CNRS UMR 8601, University Paris Descartes, F-75006 Paris, France; jean-luc.boucher@parisdescartes.fr (J.-L.B.); gildas.bertho@parisdescartes.fr (G.B.); 9 Organic Chemistry, Institute of Chemistry, Philadelphia Bldg #212, Edmond J. Safra Campus, The Hebrew University of Jerusalem, Jerusalem 9190401, Israel; isri.agranat@mail.huji.ac.il

**Keywords:** fluorescence, molecular modeling, cancer cell migration, NADPH oxidases, NO-synthases, in vivo imaging

## Abstract

Specific inhibition of NADPH oxidases (NOX) and NO-synthases (NOS), two enzymes associated with redox stress in tumor cells, has aroused great pharmacological interest. Here, we show how these enzymes distinguish between isomeric 2′- and 3′-phosphate derivatives, a difference used to improve the specificity of inhibition by isolated 2′- and 3′-phosphate isomers of our NADPH analogue NS1. Both isomers become fluorescent upon binding to their target proteins as observed by in vitro assay and in vivo imaging. The 2′-phosphate isomer of NS1 exerted more pronounced effects on NOS and NOX-dependent physiological responses than the 3′-phosphate isomer did. Docking and molecular dynamics simulations explain this specificity at the level of the NADPH site of NOX and NOS, where conserved arginine residues distinguished between the 2′-phosphate over the 3′-phosphate group, in favor of the 2′-phosphate.

## 1. Introduction

High levels of reactive oxygen and/or reactive nitrogen species (ROS or RNS, respectively) generated in tumor cells are crucial for their proliferation, invasion, and are associated with therapeutic resistance [1,2,3,4]. ROS are mainly produced by NADPH oxidases (NOX) and the mitochondrial respiratory chain while NO is generated by nitric oxide synthases (NOS). The production of ROS is also high in tumor cells as a consequence of increased metabolic rate [5]. To enable better survival, tumor cells counterbalance the high ROS levels by overexpression of antioxidant enzymatic pathways. Consequently, any change in pro-oxidant (higher ROS) or antioxidant (lower ROS) level often modifies cancer cell proliferation as observed in melanoma cells [4,6,7].

**Scheme 1:**
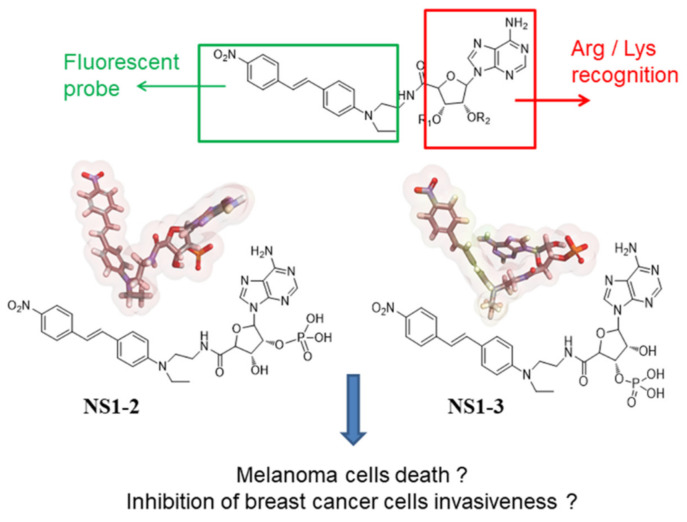
Structures of the NADPH analogues NS1 isomers, NS1-2 and NS1-3, which differ by the position of their phosphate moiety, with differences in their conformation (extended or folded) as shown by molecular modeling.

Both NOX and NOS are often overexpressed enzymes in tumor cells [6,8,9] and have different modes of regulation. However, they share the common cofactor NADPH as the source of electrons to initiate their catalysis. The NADPH binding sites of NOX and NOS comprise two subdomains, first, a chemically active one where reducing equivalents are injected into the primary electron acceptor FAD by the nicotinamide moiety of NADPH, and a second spatially separated subdomain, where recognition of conserved arginine and lysine residues of the protein binds to the 2′-phosphate group of NADPH [10]. We have designed and synthesized a fluorescent NADPH analogue called nanoshutter NS1 (Scheme 1) in which the recognition motif of the adenosine monophosphate moiety of NADPH was conserved (red box) and the nicotinamide pyrophosphate moiety of NADPH was substituted with a chromophore (green box). 

This chromophore consists of an (*E*)-stilbene substituted by an acceptor *para*-nitro group at one end and by a *para*-tertiary amine donor group at the other end, making this chromophore a push–pull moiety unable to deliver the electrons required for NOX or NOS catalysis. NS1 was synthesized as a mixture of two constitutional isomers bearing the phosphate group at positions 2′ and 3′ of the ribose moiety [10]. As expected from its design, NS1 is an inhibitor of the activity of constitutive recombinant NOS. It has been shown to reduce NO formed in aortic rings, and inhibit angiogenesis in a model of VEGF-induced endothelial tubes formation. Moreover, NS1 induces cell death by inhibition of NOX in melanoma cells without impeding the growth of healthy melanocytes [6,11]. Moreover, NS1 has interesting fluorescent properties, being non-fluorescent in aqueous solutions and becoming fluorescent upon binding to e/nNOS [10]. NS1 also inhibits NOX2 in activated macrophages [12].

Here, we explore the specificity of NOX and NOS for each isolated isomer of NS1, carrying either a 2′-phosphate, NS1-2, or a 3′-phosphate, NS1-3. We aimed at testing whether NOS and/or NOX would distinguish between the 2′ from the 3′ isomer of NS1 in their inhibition of recombinant NOS and NOX2 C-terminal (cytosolic part, residues 300–580) activities, and their effects on the viability and migratory properties of melanoma and breast cancer cells. Additionally, we investigated the fluorescence properties of the two NS1 isomers upon binding to NOX2 in vitro and in vivo. Enhanced fluorescence properties were expected, therefore, this study aimed at providing a first of a kind fluorescent NOS/NOX inhibitor.

## 2. Experimental Procedures

### 2.1. General Methods

^1^H and ^31^P NMR spectra were recorded at room temperature on an Avance II 500 MHz spectrometer (Bruker, Wissembourg, France) using deuterated DMSO as solvent. Chemical shifts δ are reported in ppm relative to the non-deuterated solvent signal and coupling constants, J, are in Hz. We assessed the attribution of chemical shifts based on 1- and 2-dimension (TOCSY, HSQC and HMBC) NMR experiments. High-resolution mass spectra (HRMS) were obtained by ESI in time-of-flight detection mode on a LCT (Waters-Micromass) spectrometer at ICSN (Gif sur Yvette, France).

### 2.2. Materials

Arginine, hemoglobin, superoxide dismutase (SOD), catalase, (6R)-5,6,7,8-tetrahydrobiopterin (H4B), NADPH, porcine brain calmodulin (CaM) and all common salts and buffers were purchased from Sigma-Aldrich (Saint-Quentin-Fallavier, France). Antibodies against NOX1 (17772-1-AP), NOX2 (19013-1-AP), NOX3 (20065-1-AP) were purchased from Proteintech, (Euromedex, Souffelweyerheim, France) the antibody against NOX5 (ab191010) was obtained from Abcam (Paris, France). 

### 2.3. Recombinant NOX2 C-Terminal Expression and Purification

The C-terminal of human NOX2 (residues 300–580) was purchased from MyBioSource, San Diego, CA, USA. It was expressed in *E. coli* Rosetta (DE3) bacterial strain at 37 °C for 3 h. The expressed 5′ His-tagged protein had a molecular weight of 35.2 KDa. It was found in the non-soluble fraction and required inclusion body purification as detailed by the manufacturer.

### 2.4. Synthesis of NS1 and Separation of Isomers

NS1 (1 g) was synthesized by Innoverda (Villejuif, France) using a previously described protocol [11] and purified by chromatography over C18-cartridge to a single peak at (M-H)- = 653 by mass spectroscopy. The analytical separation of each NS1 isomer was performed on a HPLC system consisting of an Agilent pump connected to a Kromasil 5 µ C18 (250 × 46 mm) column and an Agilent detector set at λ = 445 nm. Data acquisition and treatment were performed with Agilent software. The solvent was a mixture of A: water + 0.1% formic acid, and B: acetonitrile + 0.1% formic acid with the following gradient: 0 to 22 min, 90% A; 22 to 27 min, 20% A; 27 to 38 min, 90% A. Flow rate was 1 mL/min and injection volumes were 20 µL. The preparative separation of the isomeric mixture was performed on a preparative pre-packed C18-grafted (50 µM, 30 g) column after preconditioning with a mixture of water/acetonitrile (95:5) containing 0.2% ammonium hydroxide solution (20%), followed by elution with a mixture of water/acetonitrile (85:15) containing 0.2% ammonium hydroxide solution (20%). Ten mL fractions were collected. Pure NS1-3 fractions were collected in fraction 11–15 and enriched NS1-2 isomer in fraction 21–23. The compounds were isolated after partial evaporation followed by lyophilization. NS1-2′-phosphate isomer (NS1-2) and NS1-3′phosphate isomer (NS1-3) were recovered ~85% and >95% pure, according to analytical HPLC, ^1^H and ^31^P NMR spectroscopy (Supplementary Figures S1–S6). Due to the higher purity of NS1-3, full identification of H-atoms of its ribose moiety is shown in Figures S4 and S5.

### 2.5. Viability of Melanoma Cells

Human A375 (CRL-1619) melanoma cells were purchased from American Tissue Culture Collection (Molsheim, France). They were grown in RPMI medium supplemented with 10% fetal bovine serum (FBS) at 37 °C in a humidified atmosphere containing 5% CO_2_. The effects of NS1 isomers on melanoma cells viability were measured by using the trypan blue dye exclusion method as previously described [6,12].

### 2.6. Viability of Breast Cells, Multiple Myeloma and Colon Cancer Cells

The human breast cancer cells-MDA-MB231 were obtained from American Type Culture Collection (ATCC, LGC Standards, Molsheim, France). U266 myeloma cells were a gift from Michel Renoir, Institut Gustave Roussy, Villejuif, France and were grown in RPMI medium supplemented with 1% FBS, HTC116 colon colorectal cancer cells were a gift from Bert Vogelstein (John Hopkins, Baltimore, MD, USA) and grown in McCoy medium supplemented with 1% FBS. The effects of NS1 isomers on these cancer cells’ viability were measured by using the trypan blue dye exclusion method as previously described [6,12] or using the crystal violet method. 

### 2.7. Melanoma Cell Migration in a Boyden Chamber

Cell migration was assessed by trans-well assay (Boyden chamber, Dutscher Issy les Moulineaux, France) according to the manufacturer’s instructions. Briefly, 100,000 cells in serum-free media with or without drug were plated in the upper chamber on membranes with a pore size of 8 µm while the lower chamber contained culture media with FBS (10%) as chemoattractant. After 5 h, remaining cells were removed from the top-side of the inserts whereas migrating cells on the bottom of the inserts were stained with Diff-Quik (ThermoFischer Scientific, Waltham, MA, USA) and all migrating cells were counted. Results are expressed as means ± SEM and represent triplicate samples from at least two independent experiments.

### 2.8. Breast Cancer Cells Migration

Breast cancer SUM159PT cell line was provided by Dr P. Benaroch (Cellular Transport and Immunity Team, Curie Institute, Paris, France). SUM159PT cells were maintained in Ham’s F12 medium supplemented with 5% FBS, 1 µg/mL hydrocortisone, 10 mM HEPES and 5 µg/mL of human insulin. Migration assays were performed using xCELLigence real-time cell analysis technology (Roche, Basel, Switzerland). The xCELLigence^®^ RTCA instrument uses noninvasive electrical impedance monitoring to quantify cell invasion and migration (CIM) using an electronically integrated Boyden chamber (CIM-Plate^®^ 16) in kinetic measurements. All data were recorded and analyzed by the TRCA software. The cell index (CI) value, which is directly influenced by cell spreading and/or proliferation, was used to measure the change in the electrical impedance divided by the background value. For cell migration, cells in serum-free medium were added to the upper well of the CIM plate, uncoated basement membrane matrix. SUM159PT cells were treated with 50 µM NS1 (mixture of isomers), NS1-2 or NS1-3 or L-NAME (100 µM) for 2 h in the absence of serum (see Figure S10). After treatment, cells were washed with DPBS, harvested with trypsin and added in serum-free media to the upper well of CIM plate. The plate was incubated for 24 h and the passage of cells through the porous membrane was measured as an impedance value, expressed as the cell index (CI).

*Western Blots:* SUM159 (aggressive breast cancer) were used to investigate the effect of NS1, NS1-2 and NS1-3 on the abundance of NOX isoforms. Cell extracts were obtained after lysis with RIPA buffer (0.5% sodium deoxycholate, 50 mM Tris-HCl; pH 8, 150 mM NaCl, 1% NP40, 0.1% SDS) supplemented with protease and a phosphatase inhibitor cocktail (Roche, Basel, Switzerland) and equal amounts of protein were loaded onto SDS-PAGE gels. After transfer onto nitrocellulose membrane, blots were incubated overnight at 4 °C with the appropriate antibody, followed by incubation with a horseradish peroxidase-conjugated secondary antibody (1/2000, Cell Signaling, Danvers, MA, USA). Bands were visualized using the Clarity™ Western ECL substrate (BIO-RAD, Hercules, CA, USA). Protein expression was quantified by densitometric analysis of the immunoblots using Image Lab software developed by Bio-Rad.

### 2.9. Statistical Analysis

Data were expressed as mean ± SEM. Statistical analysis was performed using the Kruskal Wallis test with Dunn’s post-test with GraphPad Prism 6 v6.01 (GraphPad Software, Inc., San Diego, CA, USA) or with a two-way ANOVA followed by Tukey’s multiple comparisons test (GraphPad Prism version 8.4.2).

### 2.10. Effect of NS1-2 and NS1-3 on eNOS and nNOS Activity

Full length recombinant rat nNOS and bovine eNOS were expressed in Escherichia coli cell line BL21 (DE3) and purified using CaM-Sepharose column chromatography as described previously [13]. The initial rates of NO formation were determined at 37 °C in 1-cm pathlength cuvettes (total volume of 150 µL) using the oxyhemoglobin assay for NO [14]. Usual incubation mixtures were performed in 50 mM Hepes buffer (pH 7.4) containing, 0.15 M NaCl, 5 mM DTT, 10–20 µM oxyhemoglobin, 100 U/mL each SOD and catalase, 10 µM H4B, 100 µM L-arginine, 1 mM CaCl2, 10 µg/mL CaM, 4–6 µg/mL n- or eNOS (in sample cuvette), and 0–100 µM NS1 isomers (dissolved at 10 mM in DMSO). The mixtures were preincubated for 2 min at 37 °C prior to initiation of the reactions by the addition of 10 µM NADPH to both cuvettes. The NO-mediated conversion of oxyhemoglobin to methemoglobin was monitored by repetitive scanning between 380 and 480 nm every 0.2 min on a Uvikon 941 spectrophotometer and quantitated using an extinction coefficient of 77 mM^−1^·cm^−1^ between peak at 401 nm and valley at 420 nm. Control incubations were performed in the presence of similar amounts of DMSO but without NS1 isomers. All values are expressed relative to the control and are means + S.D. from 3–4 experiments.

### 2.11. Effect of NS1-2 or NS1-3 on Recombinant NOX2 C-Terminal Activity Determined by Fluorescence Measurements

The protein (5.5 µM) was incubated with 30 µM FAD for 1 h at room temperature and 1 µL of DMSO or NS1-2 or NS1-3 (5 mM in DMSO) 5 was added to the NOX2-FAD and put in a cuvette of a Uvikon 941 spectrophotometer using a reference cuvette containing 30 µM FAD in buffer. The reaction was initiated by the addition of NADPH (25 µM final concentration); 9 consecutive spectra were recorded during 40 min in the range of 500 to 250 nm. Typical results are shown in Figure 6 and correspond to the difference ΔOD monitored at the minima observed in the wavelengths 340–350 nm at time t minus recorded at time 0, immediately after addition of NADPH. The protein was stable for about 4 h.

Fluorescence spectra were measured at a fixed concentration of the compounds (1–2 µM) in 100 mM Tris buffer pH = 7.5, 150 mM NaCl with a PTI spectrophotometer set at T = 20 °C, with an excitation wavelength at 460 nm and emission wavelengths in the range 480–700 nm; slits of 10 nm were used at both excitation and emission. The protein was pre-incubated with FAD at an equimolar ratio for 15 min on ice just before the measurement.

### 2.12. Docking and MD Simulations

The ligands NS1-2 or NS1-3 were manually docked into the binding site of NADPH at the eNOS reductase domain previously obtained by homology modelling [15] with the rat nNOS-reductase domain as a template [16]. The coordinates of the co-crystallized NADPH molecule were used to correctly superimpose the sugar-phosphate-adenine group of the isomers and NADPH. Moreover, the nitro group of the isomers was positioned in order to mimic the stacking of the nicotinamide group with the isoalloxazine moiety of the FAD molecule in the active site of eNOS.

### 2.13. Molecular Docking of the Ligand (NS1) to NADPH Oxidase-2 NOX2 (gp91^phox^ DH Domain) and Molecular Dynamics (MD) Simulations of the Resulting Docked Complex

The crystal structure of the C-terminal region (spanning the amino acid residues 385 to 570) of human gp91^phox^ (NOX2) protein was available from the Protein Data Bank (PDB 3A1F). First, the human sequence of the hNOX2 (code entry: P04839) was aligned with the sequence of the N-terminal part of CsNOX5 (code entry: Q96PH1) using the Align Sequences module of Discovery Studio 2020 v18 (Biovia, Dassault System, Paris, France). The sequence alignment indicated homology rates of 63.5%. The next step was to copy a set of constraints derived from the crystal structure of the CsNOX5 isoform (PDB code: 5o0X) to the corresponding residues of the sequences to be modeled using the Modeler module of Discovery Studio v18. The FAD molecule was conserved. Twenty models were generated. The model with the lower energy was selected for the next step. The selected structure was optimized according to the below procedure.

First, the compound NS1 was docked into the model using the flexible docking protocol of Discovery Studio v18. The resulting complex was then submitted to a 100 ns molecular dynamics simulation using the GROMACS 2018 package. A clustering (RMSD cutoff: 2 Å) was finally applied to extract the most representative structure of the simulation. The structure was validated using the Ramachandran plot generated by the RAMPAGE program. This plot indicates high quality stereochemical parameters with a very good distribution of φ and ψ angles. Indeed, more than 98% of the residues were located in the favored and allowed regions (Supplementary Figure S11). For the MD simulations of the system formed by the DH domain of NOX2, the cofactor FAD, and NS1-2 or NS1-3 bound to NOX2, each complex was centered in a cubic box with an edge length of 80 Å; the box was subsequently filled with water using the Simple Point Charge (SPC) model and the system was made neutral using the Genion tool of the GROMACS package [17] (salt concentration of 0.15 mol L^−1^). The resulting system was then subjected to energy minimization (200 steps of steepest descent followed 200 steps of conjugate gradient until the maximum force was smaller than 1000.0 kJ mol^−1^·nm^−1^) to remove steric clashes and to correct inappropriate geometry. Then, an equilibration step was applied to equilibrate the solvent and ions around the complex. Equilibration was carried out in two phases. The first was conducted in an NVT ensemble (N, constant number of particles, V, volume, and T, temperature) to reach a temperature of 310 °K using a velocity rescaling thermostat. The second phase was conducted in an NPT ensemble to equilibrate the system under 1 atm pressure using the v-rescale thermostat and the Parrinello-Rahman barostat. All equilibration phases were carried out for 2 ns. Upon completion of the equilibration step, the position restraints of the entire system were released for 20 ns production of molecular dynamic simulation (MDS) runs.

### 2.14. In Vivo Experiments

Female nude mice (Hsd Athymic Nude-Foxn1nu, Envigo, Gannat, France, 8 weeks old), were injected by intra-peritoneal (IP) route with NS1 (40 mg/kg) or with the vehicle (PBS 1X). The in vivo fluorescence was detected with an IVIS camera and monitored as a function of time. The peak of fluorescence was detected after 2–3 h and decreased afterwards, some signal was still detected after a day. Tissue fluorescence acquisitions were performed with the appropriate settings for NS1 (excitation: 465 nm, back-ground excitation: 415 nm and emission: 620 nm, acquisition time: 2 s). The fluorescence image was merged to a visible light image to delineate regions of interest (ROI) that perfectly fit to tissue shape. After subtraction of autofluorescence (time 0), ROI specific-NS1 fluorescence was expressed as ph/s/cm^2^/sr (M3 Vision software, version 1.1.2.26170, Biospace Lab, Nesles-la-Vallée, France).

### 2.15. Western Blots

Tissues were grounded in RIPA 1X buffer, supplemented with protease (cocktail of protease inhibitors (1/200, P8340, Sigma-Aldrich, Saint Quentin Fallavier, France) and phosphatase inhibitors (1% PMSF, 1 mM NaF, 100 µM NaVO3, 40 mM β-glycerophosphate), using Precellys homogenizer and ceramic beads (Bertin Technologies, Montigny-le-Bretonneux, France). Total protein concentration was determined with Bio-Rad protein assay. The WB were performed as described above. The mouse anti-NOX2 antibody was used in the following conditions: 1/1000, (PA1667, Boster Bio, Pleasanton, CA, USA) and HRP secondary antibody 1/3000 (4030-05, Southern Biotech). NOX2 was detected with Immobilon Western Chemiluminescent HRP Substrate (Milipore, Guyancourt, France) and the PXi chemiluminescent imager (Syngene, Cambridge, UK). Relative NOX2 quantity was determined by densitometry using ImageJ software (NIH) and normalized to total transferred proteins stained with amido black reagent.

## 3. Results and Discussion

The synthesis of NS1 yielded two isomers in a 40:60 ratio. These isomers bear the phosphate group at positions 2′ and 3′ of the ribose moiety, respectively (Scheme 1) [10]. Successive passages of NS1 on a preparative C18-Kromasil MPLC column enabled the separation of the two isomers (Scheme 1). Compounds NS1-2 and NS1-3 were recovered at a purity of ~85 and >90%, respectively, according to ^1^H and ^31^P NMR and HPLC analysis (see Supplementary Materials and Supplementary Figures S1–S6). They were found to be stable for weeks at room temperature in DMSO by ^1^H NMR. 

### 3.1. NS1-2 and NS1-3 as Fluorescence Probes In Vitro

We tested whether NS1 becomes fluorescent by binding to NOX. To do so, we used the recombinant human NOX2 C-terminal (residues 300–580) for a challenging expression and purification thereof (see Experimental section). This protein expressed in *E. coli* was found to be relatively stable over a period of 4 h at 20 °C (see below).

**Figure 1:**
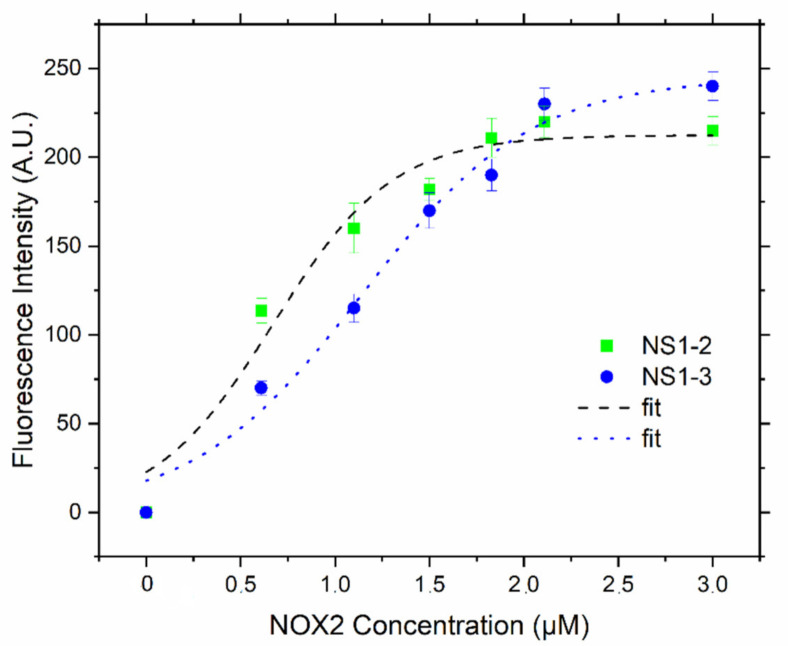
Fluorescence enhancement of NS1-2 and NS1-3 upon binding to recombinant NOX2 C-terminal; the protein was complemented with FAD (at 1/1 ratio), Excitation at 460 nm, emission recorded in the range 480–700 nm. A broad maximum emission was detected at (560–580) nm. The data were corrected for the contribution of FAD by subtracting the signal obtained with FAD in buffer at each NOX2 concentration. The data are fitted to a dose–response curve embedded in Origin 8.1 software with the following parameters: NS1-2: A_1_ (lower asymptote) = 0, A_2_ (upper asymptote) = 212 ± 11, K_D_ = 0.67 ± 0.11 µM, R^2^ = 0.965; NS1-3: A_1_ = 0, A_2_ = 244 ± 14, K_D_ = 1.14 ± 0.09 µM, R^2^ = 0.984.

Interestingly, binding to the NOX2 C-terminal of both NS1-2 and NS1-3 increased their fluorescence (Figure 1) whereas both NS1-2 and its isomer were not fluorescent in buffer. Moreover, the apparent K_D_ of NS1-2 for NOX2 was somewhat lower than that for its NS1-3 isomer, although the value should be taken with appropriate caution, due to the relative low stability of the protein. The large enhancement of fluorescence of these two isomers may serve as useful probes of (mainly) NOX and NOS, considering that an enhancement of NS1 fluorescence was observed upon binding to recombinant e/nNOS [10]. The assumption is consistent with previous cellular studies [11,12].

### 3.2. Fluorescence Properties of NS1 In Vivo

**Figure 2:**
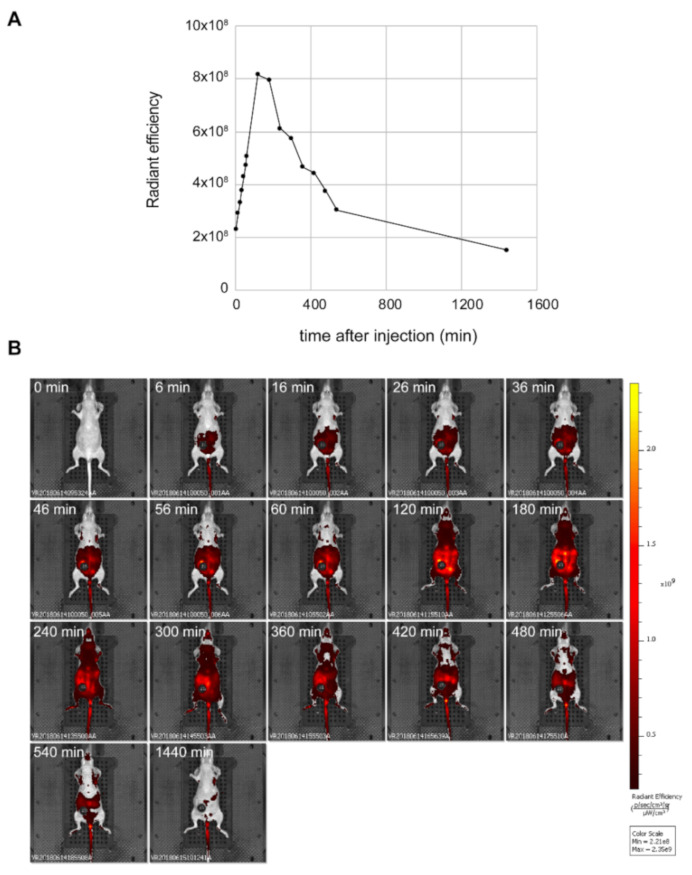
NS1 monitoring in vivo by an IVIS in vivo imaging system after a single IP injection in nude mice (NS1 concentration was 40 mg/kg in PBS). (**A**,**B**) Time course of NS1 fluorescence was monitored by in vivo imaging (the excitation was at 465 nm and the emission at 620 nm) by IVIS for 24 h.

These experiments called for testing NS1 imaging properties in vivo. As both NS1-2 and NS1-3 cannot be distinguished in terms of fluorescence yield (Figure 1), we used NS1, the mixture of the two isomers. To do so, we administrated NS1 to nude mice by intra-peritoneal injection. As it can be clearly seen in Figure 2, the fluorescence gradually built up with time and reached a plateau after 2–3 h, where the fluorescence intensity was about four-fold higher than the intensity monitored at t = 0. Further monitoring showed a slow decrease in the fluorescence up to 24 h. As the fluorescence of NS1 is due to the chromophore part of the molecule, these data indicated that NS1 chromophores remained relatively long-lived in vivo.

**Figure 3:**
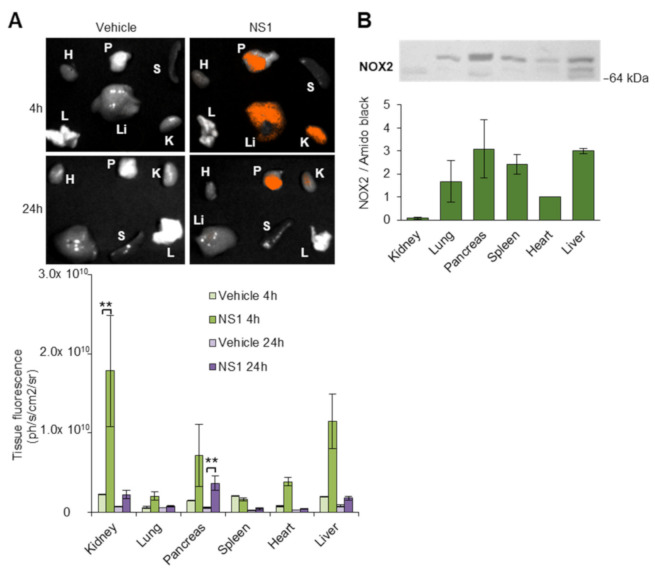
(**A**) NS1 fluorescence (excitation at 465 nm, emission at 620 nm) in selected organs of the mice after sacrifice at 4 h and 24 h post-IP relative to vehicle alone and their quantification. (**B**) NOX2 levels at 4 h in these organs determined by Western blots and their quantification relative to amino black presented in Supplementary Figure S7. The staining with amido black of the total proteins transferred onto the nitrocellulose membrane is used to normalize the levels of the protein of interest (i.e., NOX2) in each tissue analyzed. This normalization was used because any housekeeping proteins, such as β-actin or β-tubulin, show variable levels between tissues. Statistical significance indicated by stars (** *p*-value < 0.01) was evaluated by a two-way ANOVA followed by Tukey’s multiple comparisons test. A significant accumulation of NS1 was observed in the kidney after 4 h post-injection while at 24 h post-injection, NS1 fluorescence was significantly increased in the pancreas. No significant fluorescence of NS1 was noted in all other tissues analyzed.

Moreover, after sacrificing the animals at 4 h or 24 h, Figure 3 shows that the fluorescence was concentrated in certain organs such as the kidney, the liver and the pancreas, with little fluorescence in the lung, heart or spleen. The abundance of NOX isoforms is not homogeneous, rather, it is concentrated in certain organs [8]. We detected NOX2 by Western blots in the organs with enhanced fluorescence.

At 4 h post-injection, NS1 mainly accumulated in the kidney where NOX4 is expressed [18]. This result is consistent with NS1 inhibition of overexpressed NOXs [6]. We also observed some NS1 in the liver at 4 h, in agreement with NOX2 expressed in hepatocytes [8]. It is difficult to explain why NS1 was not found in the lungs and in the spleen at 4 h as these organs express NOX isoforms, in particular NOX3 and NOX5. The most remarkable accumulation of NS1 was found in the pancreas at 24 h, an organ in which NOX2, NOX4, DuOX2 and e/nNOS are expressed. NOX2 was assessed as the major NOX isoform in human islet cells, with the contribution of NOX4 in glucose-stimulated insulin secretion [19], NOX2 exerts a negative modulator role of the secretory response, reducing cAMP/PKA signaling secondary to ROS generation in pancreatic β-cells [20]. Thus, the accumulation of NS1 in the kidney and pancreas may be due to increased levels of NOX and e/nNOS in these organs. 

As NS1-2 and NS1-3 were both fluorescent-bound to NOX2, we further explored their specificity for NOX and NOS in cellular and in vitro studies. 

### 3.3. Cellular Studies

**Figure 4:**
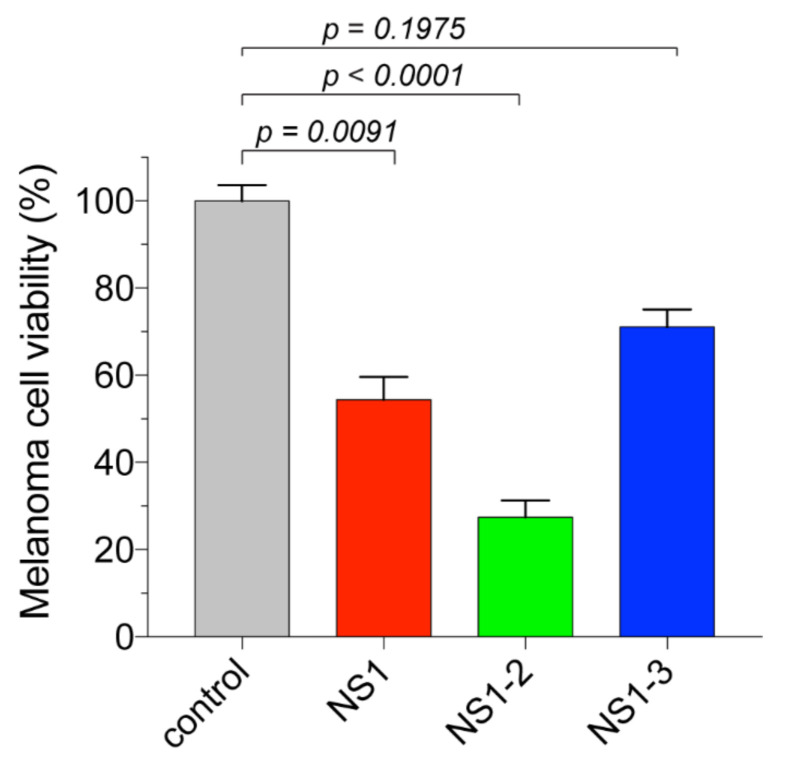
Effects of 30 µM NS1 (as a 40:60 mixture of isomers) and NS1-2 and NS1-3 on A375 melanoma cells viability after 48 h of culture. Representative data of 3 experiments in triplicate. The mean values of 5 experiments at 72 h are the following: NS1: 69 ± 4%, NS1-2: 59 ± 7%, NS1-3: 66 ± 11% (data not shown).

The ability of the two separated isomers to induce melanoma cell death was tested. Previous studies have shown that the effects of NS1 on melanoma cell viability were mainly dependent on NS1 inhibition of NOX1/2/4 [6,11]. Figure 4 shows that only 29 ± 2% of the melanoma cells survived treatment in the presence of 30 μM NS1-2 for 48 h. By contrast, NS1-3 was less efficient and 60 ± 2% melanoma cells survived. Consistently, NS1, added as a mixture of isomers, displayed a mean efficacy and reduced the cell viability to 48 ± 2% as previously reported. Kinetic studies showed that a decrease in ROS levels was observed in parallel with the inhibition of NOX1, NOX2 and NOX4 and with a cell viability decrease in multiple melanoma cell lines and cells from patients upon treatment with NS1 [6].

In contrast to A375 and other melanoma cells [6], NS1 did not reduce the viability of the other cancer cell lines assayed by trypan blue or crystal violet staining: U266 (myeloma), HCT116 (colon cancer) and aggressive breast cancer cells (MDA-MB-231), cells (see Supplementary Figure S8). Therefore, the decrease in melanoma cell viability, tested on different cell lines and primary cells from patients seems to be specific to melanoma cells among the four cancer cell lines tested [6,11].

**Figure 5:**
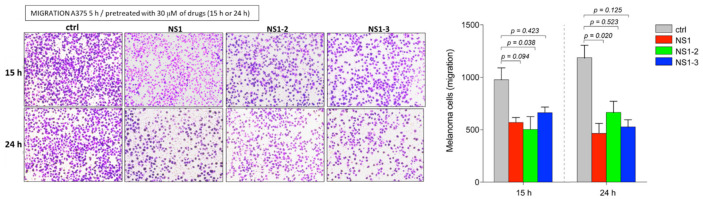
Migration of A375 cells treated or not with NS1. **Left** panel: Serum-stimulated migration assays were performed in Boyden chambers. A total of 100,000 A375 melanoma cells were incubated with NS1 or its separated isomers (30 µM) or with control for 15 h or 24 h. Then, the pre-treated cells were plated in the upper chamber in serum-free medium, while the lower chamber contained culture media with 10% serum to stimulate migration for 5 h. Representative images of the lower surface of membranes are shown; the two incubation times were expected to yield similar results due to a fast penetration (within minutes) of NS1 in cells, and likely reflect the experimental variation. **Right** panel: the mean migration of the cells is shown in triplicates from two independent experiments; error bars, SEM. *p*-values indicate the difference between the migration of control cells and the treated cells and were calculated by the Kruskal Wallis test with Dunn’s post-test (GraphPad Prism).

**Figure 6:**
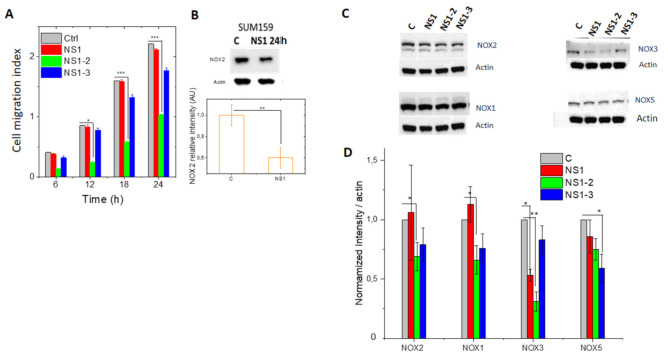
(**A**) Effect of 50 μM NS1 (mixture of isomers), NS1-2, NS1-3 on the migration of SUM159 breast cancer cells, see also Supplementary Figure S10; (**B**) Western blot analysis of NOX2 in SUM159 cells treated or not with NS1 (50 µM) and their quantification after 48 h treatment; (**C**,**D**) Representative Western blots of NOX2, NOX1, NOX3 and NOX5 in SUM159 cells treated or not with NS1 or its isomers for 48 h and their quantification.

Overexpression of multiple NOX isoforms, including NOX2, NOX4 and NOX5, as well as e/nNOS and iNOS were reported to enhance breast cancer cells tumorigenesis and invasion [4,8,9,21,22]. Indeed, SUM159 cells and other aggressive cells such as MDA-MB-231 cell lines exhibited significant levels of NOX5, NOX4, NOX2 and nNOS mRNA (Supplementary Figure S9). Therefore, we tested the effect of the 2′- and 3′-phosphate isomers of NS1 in migration assays with both melanoma A375 (Figure 5) and breast SUM159 tumor cell lines (Figure 6), and analyzed the level of various NOX isoforms by Western blots.

The migration assay showed a significant decrease in melanoma cells migration when treated with 30 µM NS1 for 24 h. The isomers 2′ or 3′ induce a similar decrease in migration to NS1 within experimental error. In these conditions of treatment, NS1 reduced the levels of ROS formed by multiple NOX isoforms found in melanoma cells, including NOX1/2 and NOX4 [6].

In an additional assay using the xCELLigence^®^ system with an integrated electronic Boyden chamber (detailed in the Experimental section), the migration capacity of the aggressive breast SUM159 cancer cells was determined by a change in the impedance of a membrane through which the cells migrated. As shown in Figure 6, NS1-2 significantly decreased breast tumor cell migration by 71 ± 2% and 55 ± 3% after 12 h and 24 h, respectively. The 3′-isomer NS1-3 only reduced the SUM159 cell migration by 12 ± 3% and 20 ± 4% after 12 h and 24 h, respectively (see also Supplementary Figure S10 for raw data and details of the difference in migration of each isomer relative to the control as a function of time). Moreover, Western blot analysis showed a decrease of 40% in NOX2 levels in NS1-treated SUM159 cells during 24 h (Figure 6B). Treatment of these breast cancer cells with 100 µM L-NAME, a NOS inhibitor, gave similar results as NS1-2 did (Supplementary Figure S10). We further tested whether NOX2 and other NOX abundance in SUM159 cells was affected by 48 h treatment with NS1 or its isomers. The level of NOX3 was most decreased by NS1-2 (50 µM), with smaller effects on NOX2 and NOX1 levels. The level of NOX5 was minimally modified by NS1-2, and NS1-3 seemed to be more effective.

Taken together, melanoma and breast cancer cells migration was inhibited by NS1 and its isomers. The efficacy of NS1-2 was greater than that of NS1 and NS1-3 in regard to inhibiting breast cancer cells migration. In contrast to NS1, which only decreased melanoma cells proliferation, NS1 and NS1-2 had no effect on the proliferation of breast MDA-MB231 cancer cells, HTC116 colon cancer and U266 myeloma cancer cell lines (Supplementary Figure S8). WB analysis of SUM159 breast cancer cells showed decreased levels of NOX isoforms, in particular NOX3, NOX2 and NOX1 by NS1-2 treatment. Both NOX1 and NOX2 are affected by NS1 inhibition in melanoma and breast cancer cells. 

### 3.4. In Vitro Recombinant Enzymatic Activities Measurements

**Figure 7:**
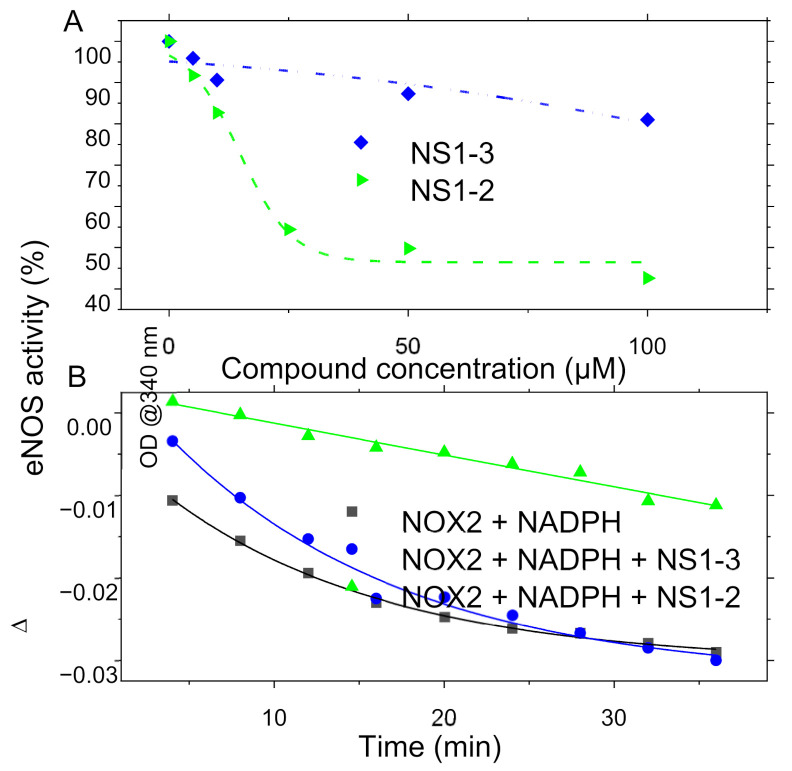
Effect of NS1 and its isomers on (**A**) eNOS activity determined by the hemoglobin assay [19]; (**B**) Representative absorption changes around 340 nm due NOX2-induced NADPH consumption using NOX2 = 2.5 µM reconstituted with 50 µM FAD, NADPH = 50 µM with or without 25 µM of NS1-2 or NS1-3 in 20 mM Tris buffer pH = 7.5, 150 mM NaCl.

To further confirm that the difference between the two isomers was indeed due to a difference of binding to NOS present in SUM159 cells, we further compared the ability of NS1-2 and NS1-3 to inhibit recombinant e/nNOS and NOX2 activity in vitro [10,11]. NS1-2 inhibited eNOS more efficiently than NS1-3 did (IC50 ~30 µM and >100 µM, respectively, Figure 7A). Similar results were obtained with recombinant nNOS (not shown). We further tested the effect of the two isomers on recombinant human NOX2 C-terminal. This protein expressed in *E. coli* was found to be relatively stable over a period of 4 h at 20 °C, but its activity was lost at more prolonged times. Nevertheless, freshly prepared NOX2 samples showed consumption of NADPH, evidenced by the decrease in the absorption of NADPH around 340 nm (Figure 7B). In the presence of NS1-2, the NADPH consumption markedly decreased while the opposite was observed in the presence of NS1-3, suggesting that NOX2 may distinguish between the two isomers, in favor of NS1-2. 

### 3.5. Docking and MD Simulations

**Figure 8:**
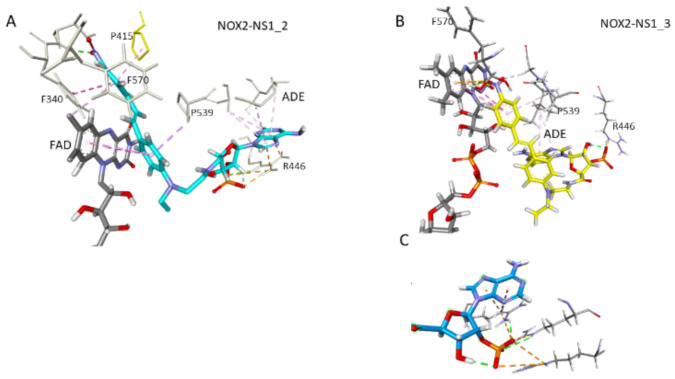
Comparison of the binding of NS1-2 (**A**, shown in cyan) and NS1-3 (**B**, shown in yellow) to a model of NOX2. For clarity, only FAD, and residues involved in the ligands binding are shown. ADE stands for adenine. (**C**) Zoom-in of the phosphate interaction with lysine/arginine residues, driving the recognition of NOS and NOX.

In order to get insight into the origin of the differences between the 2′- and 3′-phosphate isomers binding to their target proteins, NOX and NOS, we docked the two isomers in the simulated structure of NOX2, obtained by homology modelling (Supplementary Figure S11) based on PDB 3A1F (partial structure of human NOX2) and 5O0X [23] and on the structure of the reductase domain of eNOS, previously obtained by homology modelling based on PDB 1TLL [16,23]. Isolated NS1-2 in solution adopted an extended conformation with its adenosine moiety away from the chromophore (Scheme 1). NS1-2 bound to NOX2 via its 2′ phosphate moiety, forming a strong H-bond with R446 (Figure 8A). The adenosine moiety was held extended by interactions with A409, L444 and A451. The chromophore group stacked with FAD isoalloxazine ring and experienced hydrophobic interactions with P539, F340, P415, T341 and F570. Isolated NS1-3 in the solvent was mainly folded due to adenine folding over the chromophore (Scheme 1). The same holds true when bound to NOX2 via its 3′ phosphate moiety to R446.

NS1-3 adenine folding back was stabilized by I411, an H-bond between the nitro group and G412 and induced a deviation of the chromophore from planarity (Figure 8B). This folded structure likely created a steric hindrance for binding to the protein. Accordingly, the NS1-3-NOX2 complex exhibited a lower interaction energy as compared to the NS1-2-NOX2 complex (−44 versus −55 Kcal/mole, respectively). 

**Figure 9:**
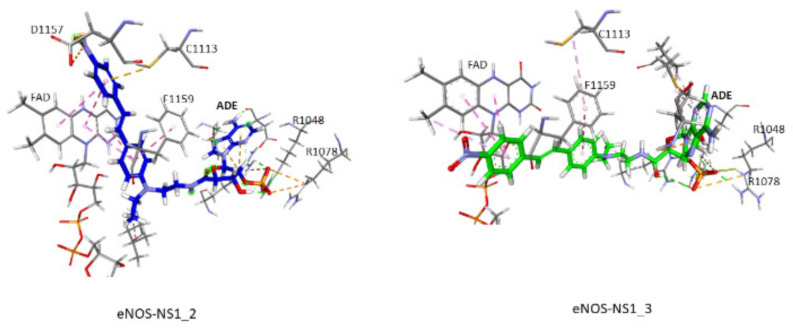
Binding of NS1-2 (**left**, shown in blue) and NS1-3 (**right**, shown in green) to eNOS reductase domain; the main residues involved in the interactions with eNOS are labeled. ADE stands for adenosine.

Binding to eNOS was mediated by the strong interactions of the 2′- and 3′-phosphate groups of NS1-2 and NS1-3 with the conserved arginine residues R1078 and R1048 (Figure 9). Constrained by the strongest electrostatic arginine–3′-phosphate interaction, the adenine had rearranged. This rearrangement, at least in part, drove the chromophore orientation and further interaction of the chromophore with FAD. NS1-3 was oriented in a parallel conformation relative to the plane of FAD isoalloxazine rings rather than being perpendicular as NS1-2 was. Consequently, the adenine folded back onto the chromophore, held in place by hydrophobic interactions, a folded structure also observed in photoactive NADPH analogue [24] carrying a 3′-carboxylate instead of a 3′-phosphate group [25]. It was reported that NOX2 binds a NADPH mimetic where a bulkier azidonitrophenyl-aminobutyryl group substituted its 3′-OH group with a decrease of only 1.9 folds as compared to NADPH itself [26], indicating little effect of steric hindrance at the 3′-position in the presence of a 2′-phosphate group linked to the flexible nicotinamide. The relative rigidity of the amide group linking the 2′-phosphate–AMP group to the chromophore also contributed to the good insertion of NS1-2 within the NADPH site of eNOS and NOX2.

## 4. Conclusions

We showed that NOX2 and e/nNOS distinguished between NADPH analogues bearing a 2′- or a 3′-phosphate group. This distinction was observed in vitro by enhanced physiological responses depending on NOS or NOX2, inducing a specific cell death of melanoma cells. We observed a clear reduction in the migration capacity of breast cancer cells treated with NS1-2. The same holds true for melanoma cells, but NS1, NS1-2 and NS1-3 were equally potent in decreasing their migration capacity, within experimental error. 

It is interesting to note that NS1-2 inhibition of SUM159 breast cancer cells invasion was most efficient between 6 and 12 h and declined afterwards by 2.5-fold (Figure 5 and Supplementary Figure S10). NS1-3 also decreased this activity but only by 1.5-fold. As the activity of NS1 towards NOX2 in a cell-free assay was lost when its phosphate moiety was substituted by an OH group [12], this result suggests that the phosphatase(s) found in breast cancer cells, likely has some specificity for 2′ over 3′ phosphate. Indeed, phosphatase activity can be enhanced by the decreased ROS levels due to NOX inhibition [27]. Moreover, recent work has shown that local NADP(H) levels are regulated by a phosphatase targeting the 2′-phosphate of NADPH called Nocturnin, linking metabolism, and redox status with the circadian clock [28]. Thus, the distinction of 2′-phosphate from 3′-phosphate could be shared by enzymes such as NOX and NOS and possibly some phosphatases [29] involved in a selective catalysis [30]. Accordingly, we show here a clear inhibition of recombinant n- and eNOS- and NOX-dependent activities by NADPH analogues bearing a 2′-phosphate moiety. The calculated structures of NS1-2 bound to eNOS and NOX2 supported these conclusions.

In vivo studies showed significant accumulations of NS1 and NOX2 in the pancreas at 24 h. NOX2 [20], DuOX2 [8] and eNOS and nNOS [31] are expressed in the pancreas, with NOX and NOS contributing to normal and impaired beta-cells functions. Further in-depth analysis of NOX1/NOX2/NOX4 profiles in mouse pancreas may shed more light on the role of NOX in metabolism and test whether NS1 could act as a metabolic fluorescent marker. 

Taken together, this work highlights the highly valuable properties of NS1-2, that is, it combines in vivo fluorescence in selected organs, presenting a relatively long-lived pharmaco-kinetics profile while being non-fluorescent in aqueous buffer, with improved targeting of NOS and NOX in a cellular context. Work is in progress to test whether the interesting pharmacological activity of NS1-2 in vitro can also be found in vivo.

## Data Availability

Data is contained within the article and supplementary material.

## Supplementary Materials

The following are available online at https://www.mdpi.com/article/10.3390/antiox10050723/s1, Experimental procedures and Figures S1–S11 are gathered in the Supplementary Material. Figures S1–S6 describe the separation of the two isomers and their chacracterization by HPLC and NMR, Figure S7 illustrates illustrates the amido black staining, as loading control of the WB anti-NOX2 in Figure 3B, Figure S8 presents cell viabilities of cancer cells treated or not with NS1 or its separated isomers using trypan blue, Figure S9 presents NOX and NOS QPCR, Figure S10 cell migration raw data and Figure S11 shows a plot attesting the quality of our NOX2 model.

## Abbreviations

FAD	Flavin adenine dinucleotide
L-NAME	N(ω)-nitro-L-arginine methyl ester
MD	molecular dynamics
NOX	NADPH oxidase
NOS	NO-synthase
NS1	nanoshutter 1 [11]
