# Supplementary material for: Distinction between 2′- and 3′-Phosphate Isomers of a Fluorescent NADPH Analogue Led to Strong Inhibition of Cancer Cells Migration
Source: Antioxidants (Basel). 2021 May 4;10(5):723. doi: 10.3390/antiox10050723 (PMC8148004; doi:10.3390/antiox10050723)

Electronic Supplementary data

Distinction between 2'- and 3' - phosphate isomers of a fluorescent NADPH analogue led to strong inhibition of cancer cells invasion.

Raoul Manuel[a‡], Michelle de Souza Lima[a ‡], Sébastien Dilly [a, ‡], Sylvain Daunay[b], Patricia Abbe[c], Elodie Pramil[a], Stéphanie Solier[d], Fabienne Guillaumonde[e], Sarah-Simha Tubiana[e], Alexandre Escargueil[a], João Antonio Pêgas Henriques [f,g], Nathalie Ferrand[a], Irène Erdelmeier[b], Jean-Luc Boucher[h], Gildas Bertho[h], Israel Agranat[i], Stéphane Rocchi[c], Michèle Sabbah[a] and Anny Slama Schwok[a*]

Table of Contents

I-Chemistry

Separation of isomers p2

II-Supplementary Figures

Figure S1: HPLC profiles of purified NS1-2 and NS1-3. P4

Figure S2: ^{1}H spectra of isolated NS1-2. P5

Figure S3: ^{31}P NMR spectra of isolated NS1-2. P6

Figure S4: ^{1}H NMR spectra of isolated NS1-3. P7

Figure S5: TOCSY NMR spectra of isolated NS1-3. P8

Figure S6: ^{31}P NMR spectra of isolated NS1-3. P9

Figure S7: NOX2 Western blot in different mice organs and amido black gel p10

Figure S8: Cell viability of U266 (myeloma) and HTC116 **(**colon cancer**)** treated cells with NS1, NS1-2 or NS1-3; viability of breast cancer cells MDA-MB231 treated with NS1 p10

Figure S9: Abundance of NOS and NOX isoforms mRNAs in MDA231 cells. P11

Figure S10: Difference in cell migration relative to control of treated cells with NS1-2 or NS1-3 and Raw cell migration data P11

Figure S11: Ramachandran plots of the NOX2 model. P12

I-Chemistry

Separation of isomers

The analytic separation of each isomer was performed on a HPLC system consisting of a Agilent pump connected to a Kromasil 5µ C18 (250 x 46 mm) column and a Agilent detector set at λ = 445 nm. Data acquisition and treatment were performed with Agilent software. The solvent was a mixture of A: water + 0.1% formic acid, and B: acetonitrile + 0.1% formic acid with the following gradient: 0 to 22 min, 90% A; 22 to 27 min, 20% A; 27 to 38 min, 90% A. Flow rate was 1 mL/min and injection volumes were 20 µL.

The preparative separation of the isomeric mixture was performed on a preparative prepacked C18-grafted (50µm, 30g) column after preconditioning with a mixture of water/acetonitrile (95:5) containing 0,2% ammonium hydroxide solution (20%), followed by elution with a mixture of water/acetonitrile (85:15) containing 0,2% ammonium hydroxide solution (20%). Ten mL fractions were collected. Pure NS1-3 fractions were collected in fractions 11-15 and enriched NS1-2 isomer in fraction 21-23. The compounds were isolated after partial evaporation followed by lyophilization.

NS1-2'-Phosphate isomer (NS1-2) and NS1-3'phosphate isomer (NS1-3) were recovered ~85% and > 95% pure according to analytical HPLC, ^{1}H and ^{31}P NMR spectroscopy (Supplementary figures 1-6). Due to the higher purity of NS1-3, full identification of H-atoms of its ribose moiety is shown on Figures S4 and S5.

NMR spectra of NS1-2

^{1}H NMR (DMSO-d_6) δ 9.19 (t, 1H, NH, J = 5.5), 8.40 (s, H_{21}), 8.17 (d, $2H_{16}$, $J^{H15\text{-}H16}$ = 9.0), 8.00 (s, H_{18}), 7.77 (d, $2H_{15}$, $J^{H15\text{-}H16}$ = 9.0), 7.48 (d, $2H_{10}$, $J^{H9\text{-}H10}$ = 8.5), 7.40 (m, H_{12} + NH_2 $J^{H12\text{-}H13}$ = 15.0), 7.10 (d, H_{13}, $J^{H12\text{-}H13}$ = 15.0), 6.77 (d, $2H_9$, $J^{H9\text{-}H10}$= 8.5), 6.06 (d, H_1, $J^{H1\text{-}H2}$ = 7.6), 4.82 (m, H_2, $J^{H2\text{-}H3}$ = 4.5, $J^{H2\text{-}P}$ = 3.0), 4.46 (dd, H_3, $J^{H2\text{-}H3}$ = 4.5, $J^{H3\text{-}H4}$ = 1.7), 4.34 (d, H_4, $J^{H3\text{-}H4}$ = 1.7), 3.44-3.39 (m, $2H_6$ + $2H_7$ + $2H_{23}$), 1.07 (t, $3H_{24}$, $J^{H23\text{-}H24}$ = 7.0).

^{13}C NMR (125 MHz, DMSO-d_6) δ 170.1 (C=O), 156.2 (C), 152.4 (CH), 148.9 (C), 148.0 (C), 145.3 (C), 145.0 (C), 140.5 (CH), 134.0 (CH), 128.8 (CH), 126.2 (CH), 124.0 (CH), 123.4 (C), 120.8 (CH), 111.4 (CH), 87.5 (C_1H), 84.1 (C_4H), 75.1 (C_2H), 72.8 (C_3H), 48.5 (CH_2), 44.8 (CH_2), 36.4 (CH_2), 12.0 (CH_3).

^{31}P NMR (DMSO-d_6) δ – 0.74.

NMR spectra of NS1-3

^{1}H NMR (DMSO-d_6) δ 9.27 (t, 1H, NH, J = 5.5), 8.40 (s, H_{21}), 8.16 (d, $2H_{16}$, $J^{H15\text{-}H16}$ = 9.0), 8.07 (s, H_{18}), 7.76 (d, $2H_{15}$, $J^{H15\text{-}H16}$ = 9.0), 7.48 (d, $2H_{10}$, $J^{H9\text{-}H10}$ = 8.5), 7.40 (m, H_{12} + NH_2, $J^{H12\text{-}H13}$ = 15.0), 7.10 (d, H_{13}, $J^{H12\text{-}H13}$ = 15.0), 6.78 (d, $2H_9$, $J^{H9\text{-}H10}$ = 8.5), 5.98 (d, H_1, $J^{H1\text{-}H2}$ = 7.8), 4.69 (m, H_3, $J^{H2\text{-}H3}$ = 4.1, $J^{H3\text{-}P}$ = 7.4), 4.60 (dd, H_2, $J^{H2\text{-}H3}$ = 4.1), 4.43 (d, H_4, $J^{H3\text{-}H4}$ = 0.8), 3.41-3.35 (m, $2H_6$ + $2H_7$ + $2H_{23}$), 1.09 (t, $3H_{24}$, $J^{H23\text{-}H24}$ = 7.0).

^{13}C NMR (125 MHz, DMSO-d_6) δ 169.5 (C=O), 156.2 (C), 152.4 (CH), 148.9 (C), 148.0 (C), 145.3 (C), 145.0 (C), 140.5 (CH), 134.0 (CH), 128.8 (CH), 126.2 (CH), 124.0 (CH), 123.4 (C), 120.8 (CH), 111.4 (CH), 88.1 (C_1H), 83.3 (C_4H), 75.9 (C_3H), 72.1 (C_2H), 48.5 (CH_2), 44.8 (CH_2), 36.4 (CH_2), 12.0 (CH_3).

^{31}P NMR (DMSO-d_6) δ 0.94.

Figure S1: HPLC profiles of purified NS1-2 (top) and NS1-3 (bottom).

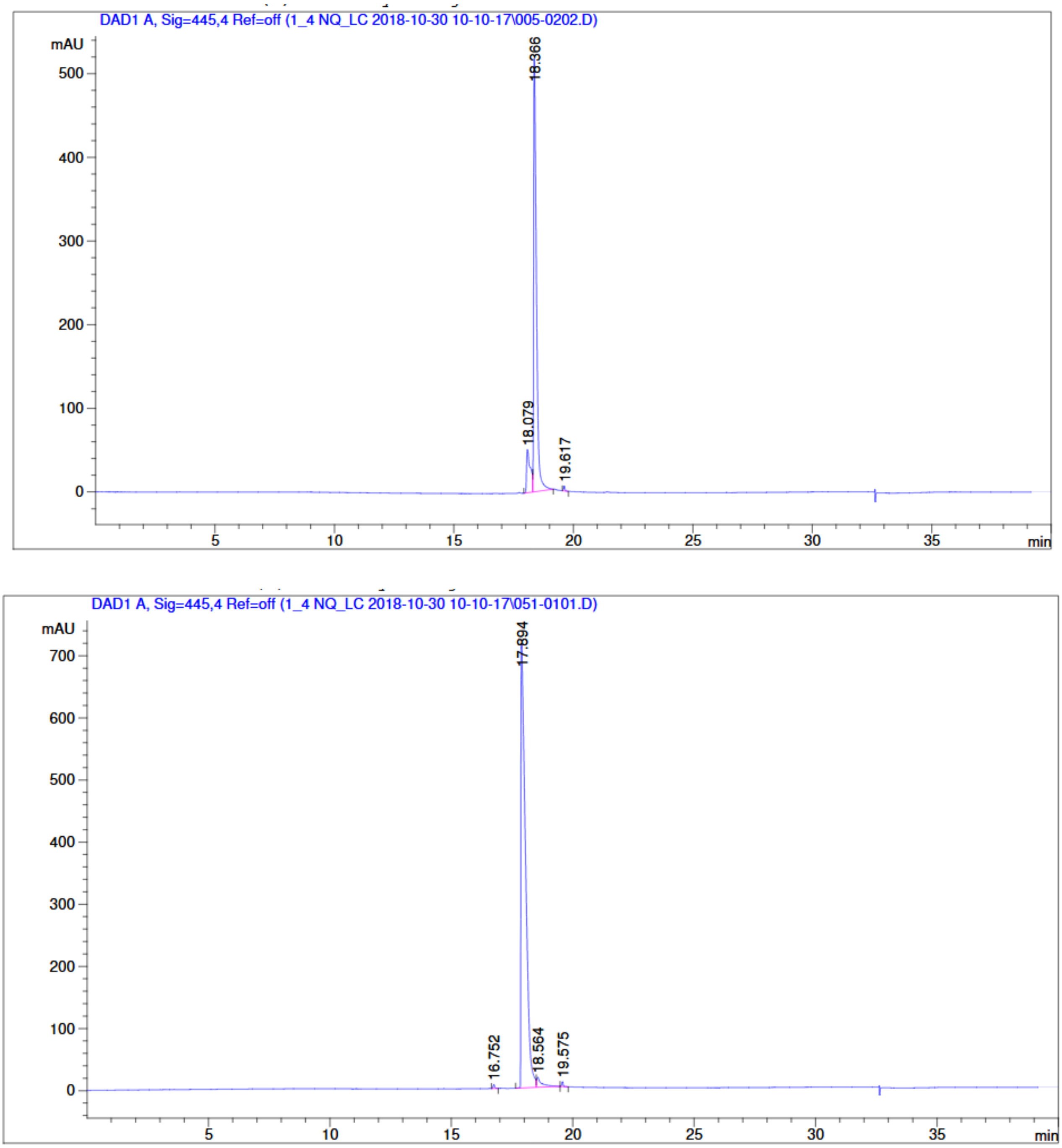

X-axis : Retention time (min). Y-axis: Absorbance at 450 nm.

Figure S2: ^{1}H spectra of isolated NS1-2. Top: 4.0-9.5 ppm region. Bottom: 6.5-4.0 ppm region.

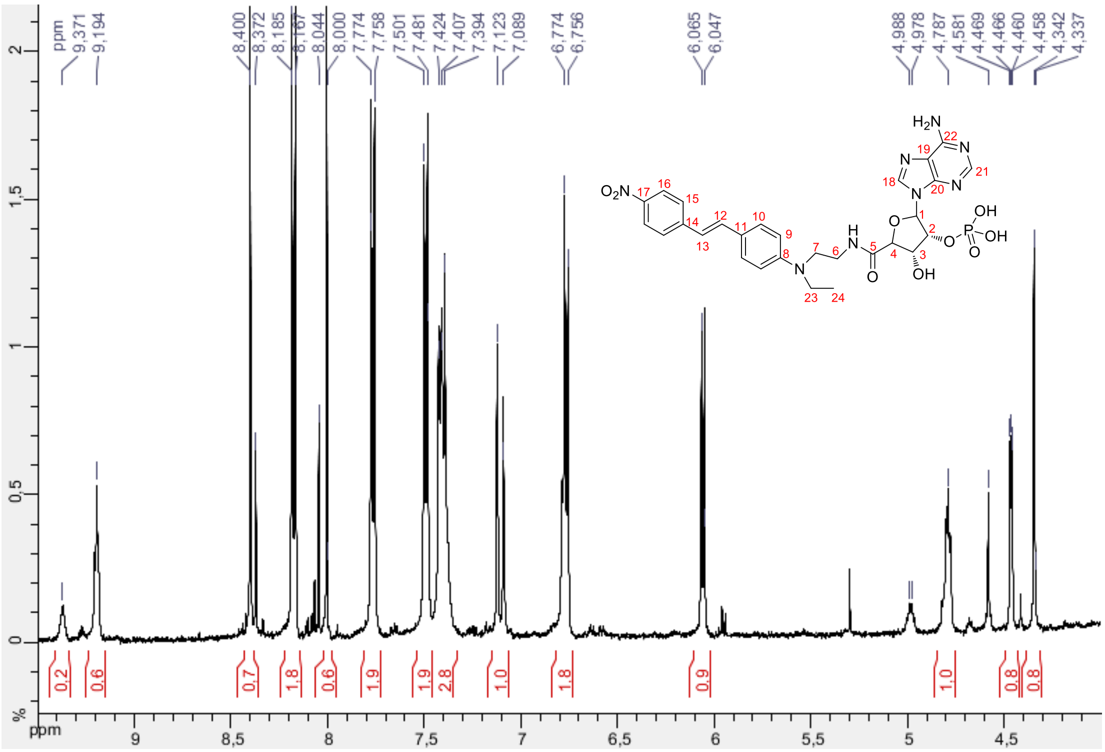

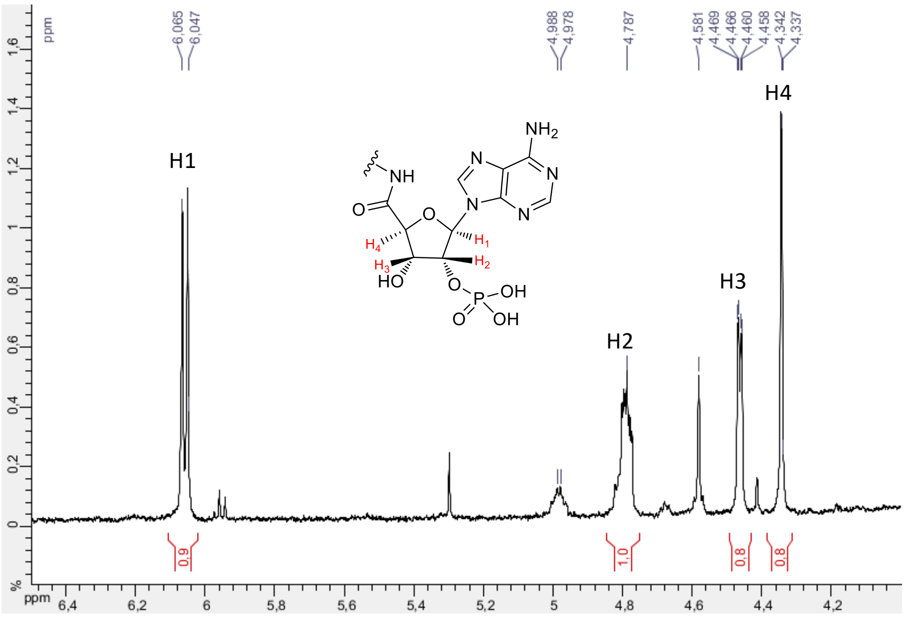

Figure S3: ^{31}P NMR spectra of isolated NS1-2.

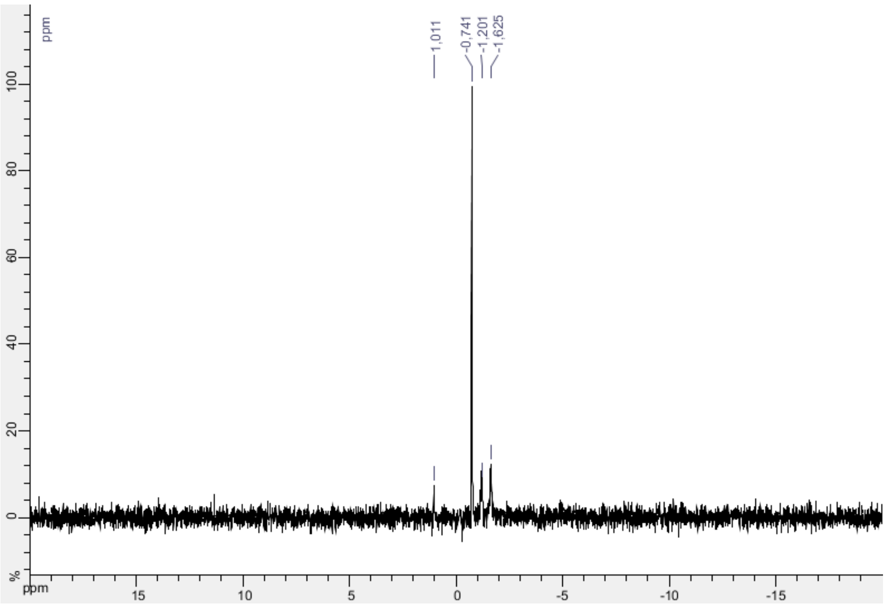

Figure S4: ^{1}H NMR spectra of isolated NS1-3. Top: 4.0-9.5 ppm region. Bottom: 6.5-4.0 ppm region.

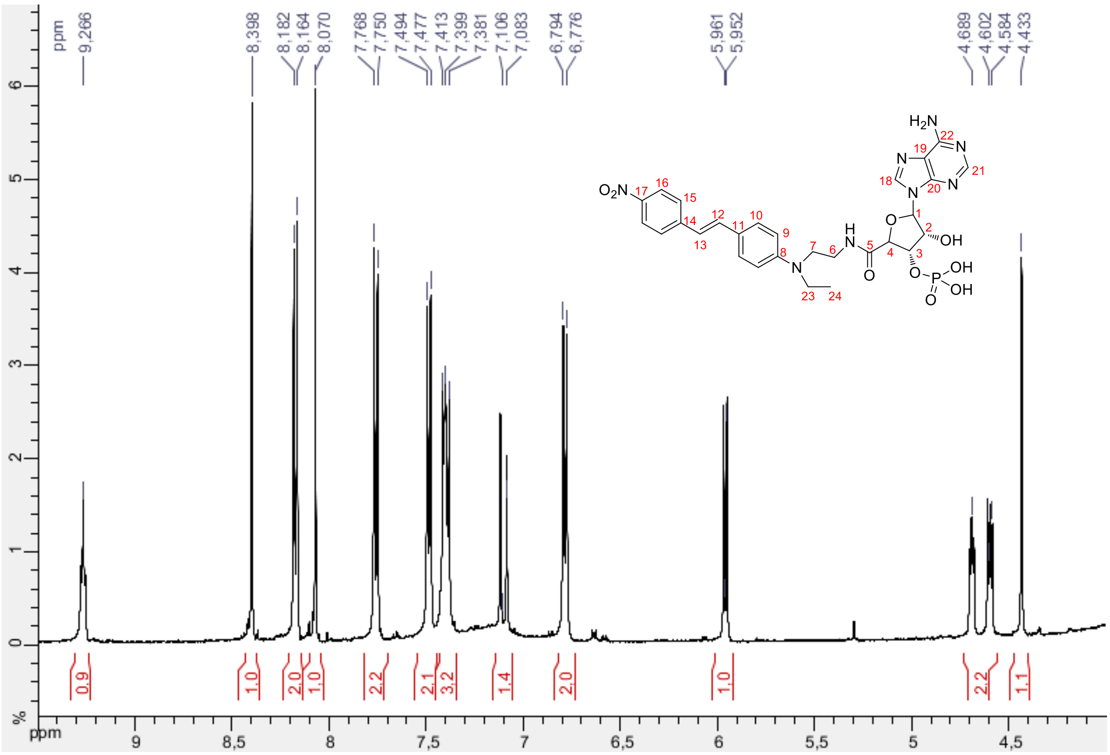

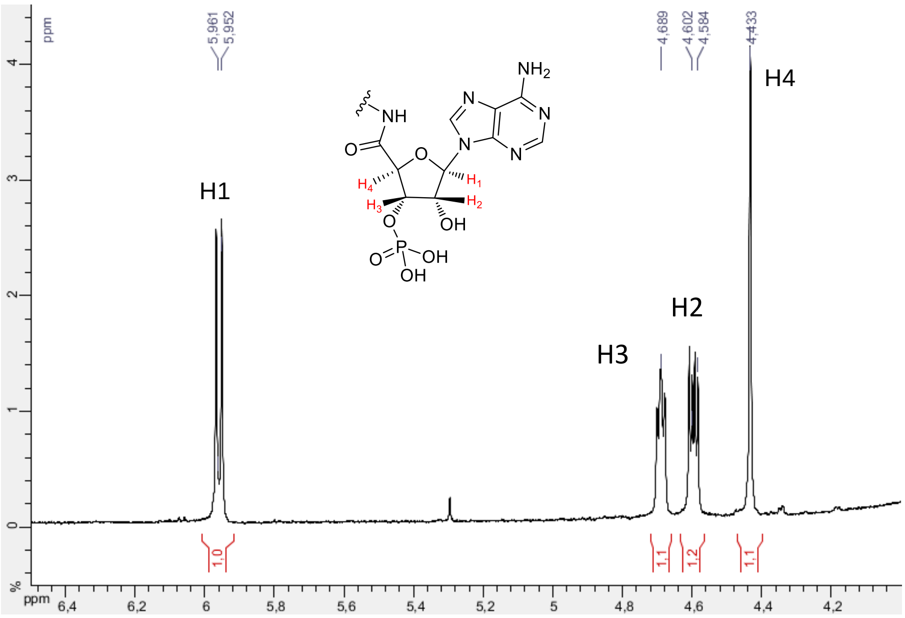

Figure S5: TOCSY NMR spectra of isolated NS1-3 (4.5-6.2 ppm region). Short and long mixing times allow clear identification of coupling constants.

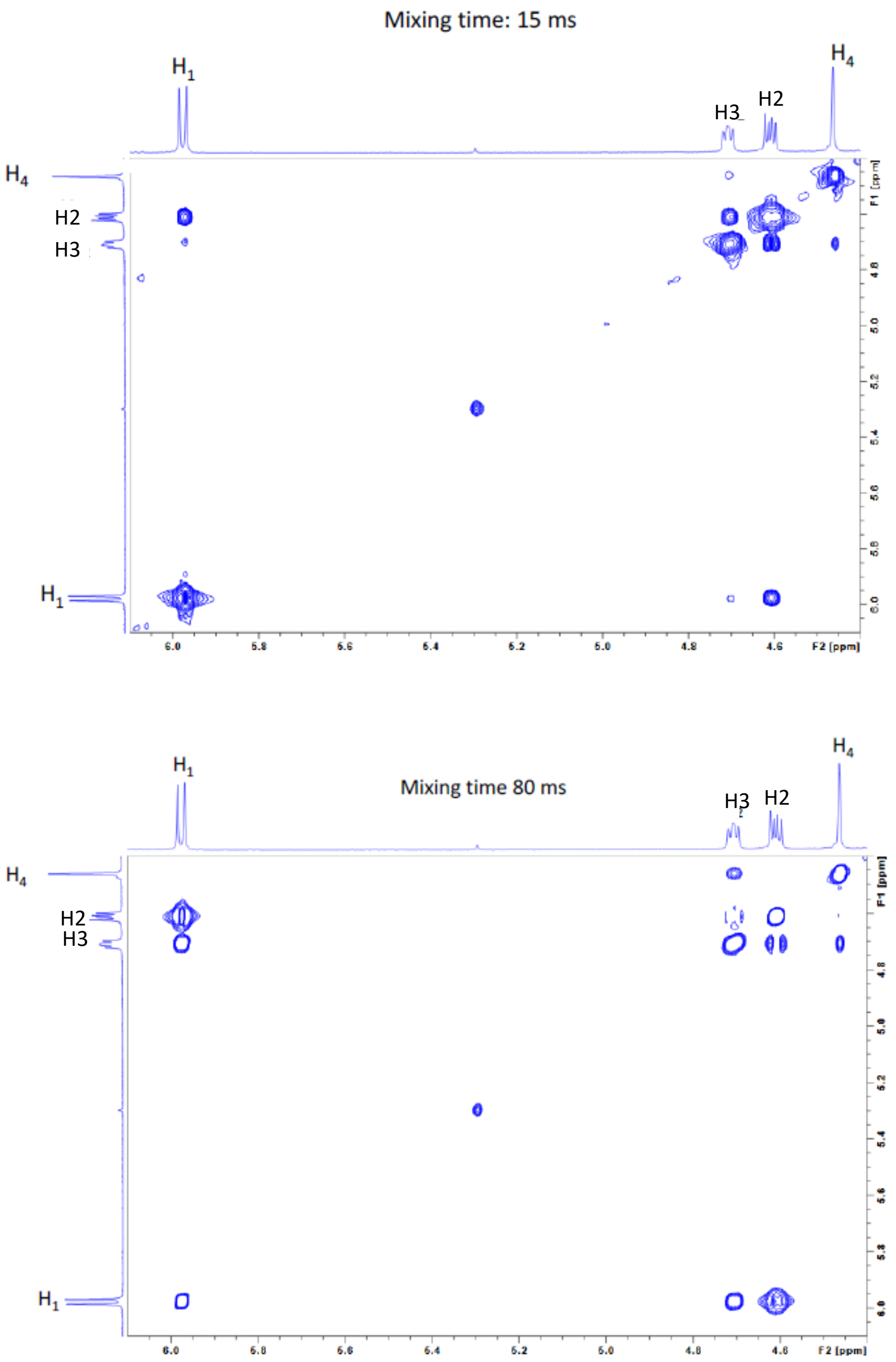

Figure S7: NOX2 Western blot in different mice organs and amido black gel

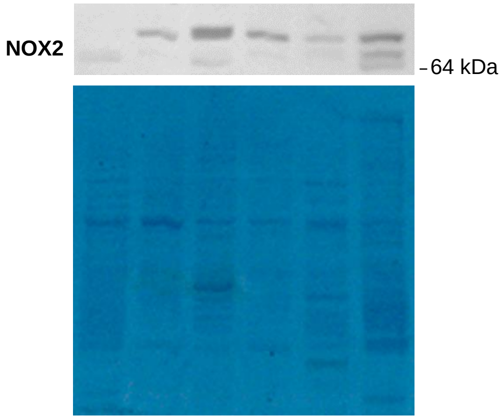

Figure S8: **Cell viability assays** (by trypan blue or crystal violet cell counting)

A: Viability at 24h and 48 h of U266 myeloma cells and HTC116 colon cancer cells treated for 24 or 48h with 50 µM NS1, NS1-2 or NS1-3 as compared to DMSO. No change of viability of treated breast cancer cells has been observed using B: MDA-MB-231 cancer cells; the cells were treated during 2h with NS1 at different doses, washed and further cultured in fresh medium for 72h, their viability was tested at 72h.

A

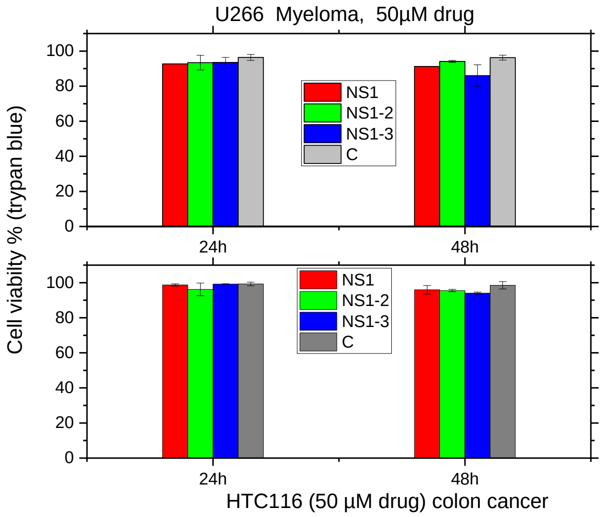

B

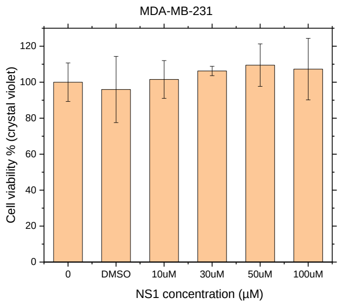

Figure S9:.**Q-PCR of NOX isoforms in MDA-MB-231 cell line**

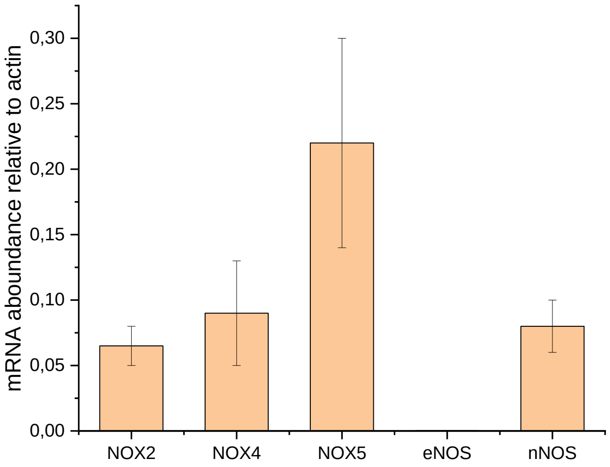

Figure S10: A-**Quantification of the cell migration difference of treated cells with NS1-2 or NS1-3 relative to control; B- Raw cell migration data**

A

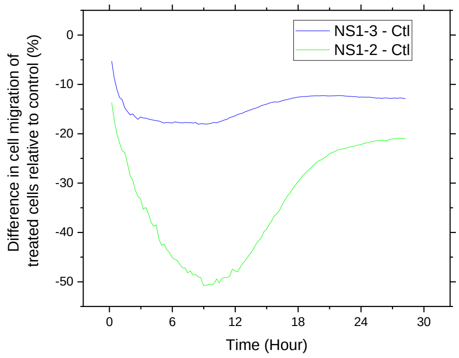

B

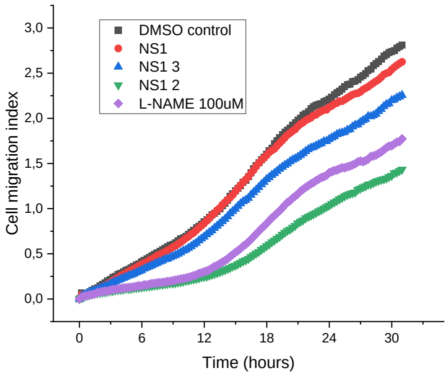

Figure S11: Ramachandran plots showing the quality of the NOX2 model obtained by homology modelling.

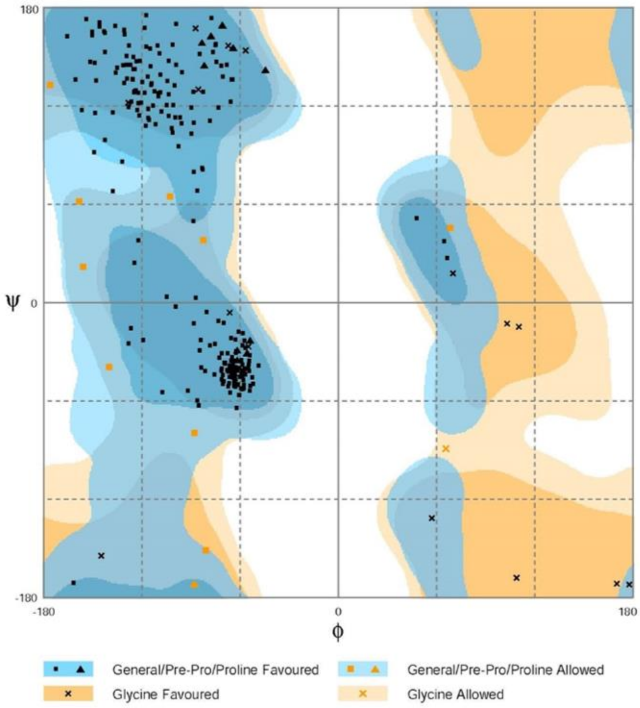

Supplement: Supplementary file 1 [file antioxidants-10-00723-s001.zip › antioxidants-1075813-supplementary.pdf]
